# Eucalyptol Inhibits Amyloid-β-Induced Barrier Dysfunction in Glucose-Exposed Retinal Pigment Epithelial Cells and Diabetic Eyes

**DOI:** 10.3390/antiox9101000

**Published:** 2020-10-15

**Authors:** Dong Yeon Kim, Min-Kyung Kang, Eun-Jung Lee, Yun-Ho Kim, Hyeongjoo Oh, Soo-Il Kim, Su Yeon Oh, Woojin Na, Young-Hee Kang

**Affiliations:** Department of Food Science and Nutrition and The Korean Institute of Nutrition, Hallym University, Chuncheon 24252, Korea; ehddus3290@naver.com (D.Y.K.); mitholy@hallym.ac.kr (M.-K.K.); reydmswjd@naver.com (E.-J.L.); royalskim@hallym.ac.kr (Y.-H.K.); ohhyeongju@gmail.com (H.O.); ky4850@naver.com (S.-I.K.); suy0411@naver.com (S.Y.O.); nsm0729@hanmail.net (W.N.)

**Keywords:** amyloid-β, apoptosis, blood-retinal barrier, db/db mice, eucalyptol, glucose, retinal pigment epithelium, tight junction

## Abstract

Hyperglycemia elicits tight junction disruption and blood-retinal barrier breakdown, resulting in diabetes-associated vison loss. Eucalyptol is a natural compound found in eucalyptus oil with diverse bioactivities. This study evaluated that eucalyptol ameliorated tight junctions and retinal barrier function in glucose/amyloid-β (Aβ)-exposed human retinal pigment epithelial (RPE) cells and in db/db mouse eyes. RPE cells were cultured in media containing 33 mM glucose or 5 μM Aβ for 4 days in the presence of 1–20 μM eucalyptol. The in vivo animal study employed db/db mice orally administrated with 10 mg/kg eucalyptol. Nontoxic eucalyptol inhibited the Aβ induction in glucose-loaded RPE cells and diabetic mouse eyes. Eucalyptol reversed the induction of tight junction-associated proteins of ZO-1, occludin-1 and matrix metalloproteinases in glucose- or Aβ-exposed RPE cells and in diabetic eyes, accompanying inhibition of RPE detachment from Bruch’s membrane. Adding eucalyptol to glucose- or Aβ-loaded RPE cells, and diabetic mouse eyes reciprocally reversed induction/activation of apoptosis-related bcl-2, bax, cytochrome C/Apaf-1 and caspases. Eucalyptol attenuated the generation of reactive oxygen species and the induction of receptor for advanced glycation end products in Aβ-exposed RPE cells and diabetic eyes. Eucalyptol may ameliorate RPE barrier dysfunction in diabetic eyes through counteracting Aβ-mediated oxidative stress-induced RPE cell apoptosis.

## 1. Introduction

Retinitis pigmentosa (RP) is an outer retinal degenerative disorder in which rod photoreceptors and retinal pigment epithelial (RPE) cells are damaged, leading to peripheral and night-time vision loss [1,2]. Diabetic retinopathy (DR) is characterized by abnormal permeability of the blood-retinal barrier, pericyte loss, basement thickening and retinal neuronal abnormalities [3,4]. Ultimately, the disruption of blood-retinal barrier and leakage of the blood contents accompany severe vision loss in both RP and DR [5,6,7]. The RPE, a monolayer of end part of retina, locates between the neural retina and the choroid, involving the outer blood-retinal barrier, and supports the structural and functional integrity of the retina [8,9]. The RPE barrier takes in adherent junctions, tight junctions and transporters, [6,8]. Tight junctions are intricate complexes of both transmembrane and cytoplasmic proteins of zonula occludens-1 (ZO-1) and occludin-1 [10,11]. These junction proteins form a selective permeable barrier between adjacent RPE cells and anchor the actin cytoskeleton components, which is essential for the maintenance of visual function of eyes [3,4,12]. Under hyperglycemic conditions, the collapse of the blood-retinal barrier arises owing to disruption of tight junctions, resulting in diabetic macular edema, a major cause of vision loss [7,8,13]. Macular edema can contribute to visual loss in the RP [14]. On the other hand, hyperglycemia-associated inflammatory pathways lead to the impairment of blood-retinal barrier [15,16]. However, the involved mechanisms are mostly unknown.

Alzheimer’s disease and RP are neurodegenerative diseases stemming from the same basic cell death mechanisms [17]. Recent evidence suggests that hyperglycemia is an increased risk for developing dementia, especially Alzheimer’s disease pathologically characterized by amyloid-β (Aβ) plaque deposits and neurofibrillary tangle in the brain [18,19]. However, the cellular and molecular mechanisms by which glucose toxicity can promote pathology of Alzheimer’s disease remains unknown. It has been shown that diabetes mellitus causes an increase in Aβ peptide levels [20,21]. A potential relationship between Aβ-derived neurotoxins and retinal degeneration has been implicated in diabetes as well as aging and Alzheimer’s disease [21]. Aβ is found within RPE cells and in extracellular drusen deposits present between the RPE and Bruch’s membrane [22,23]. The accumulation of Aβ peptide in retina is responsible for the development of retinal cell apoptosis [24]. In addition, the Aβ treatment leads to pathophysiological alternations of barrier damage, apoptotic cell death and Aβ production-triggered events in an Aβ-induced retinal degeneration in mice [25]. The NLRP3 inflammasome activation is induced by Aβ via reactive oxygen species (ROS)-dependent oxidative stress, which may be responsible for RPE dysfunction in age-related macular degeneration [26]. However, the involvement of Aβ in the development of DR remains vague. While the unfavorable effects of Aβ on retinas have been broadly evaluated, Aβ-induced RPE dysfunction and barrier impairment need to be fully investigated. Defining the molecular mechanisms and signals manipulating the blood-retinal barrier and accumulation of Aβ following RPE injury will contribute to the development of new therapeutic strategies preventing and treating eye diseases.

Eucalyptol (1,8-cineole) is a natural organic compound chiefly found in eucalyptus oil, rosemary, tea tree, bay leaves and other aromatic foliage [27]. Eucalyptol exhibits multiple pharmacological effects including anti-microbial, antioxidant and anti-inflammatory activities [28,29]. Our previous studies show that multifunctional eucalyptol exerts the renoprotection through inhibiting diabetes-associated disruption of podocyte slit junctions and disjunction of renal tubular epithelial cells [30,31]. However, it is elusive whether eucalyptol ameliorates blood-retinal barrier breakdown through inhibiting accumulation and its malicious effects of Aβ in DR. The present study investigated that eucalyptol improved disruption of blood-retinal barrier through maintaining robust cell-cell tight junctions in high glucose/Aβ-exposed RPE cells and in db/db mouse eyes. In diabetic eyes was examined the mechanism(s) germane to RPE dysfunction, and the contribution of Aβ-RAGE system and oxidative stress to the blood-retina impairment.

## 2. Materials and Methods

### 2.1. Materials

Dulbecco’s modified eagle medium (DMEM, low glucose), mannitol, D-glucose and eucalyptol were provided by Sigma-Aldrich Chemical (St Louis, MO, USA). Fetal bovine serum (FBS), trypsin-ethylenediaminetetraacetic acid and penicillin-streptomycin were obtained from Lonza (Walkersvillle, MD, USA). Aβ protein was obtained from Calbiochem (San Diego, CA, USA). Mouse monoclonal antibodies of matrix metalloproteinase (MMP)-2, MMP-9, receptor for advanced glycation end products (RAGE), Aβ and cytochrome C, and rabbit polyclonal antibody of occludin-1 were supplied by Santa Cruz Biotechnology (Santa Cruz, CA, USA). Rabbit polyclonal antibody of ZO-1 was obtained from Thermo Fisher Scientific (Waltham, MA, USA). Rabbit monoclonal Apaf-1 antibody was supplied by Abcam Biochemicals (Cambridge, UK). Antibodies of mouse polyclonal cleaved caspase-9 and rabbit polyclonal cleaved caspase-3 were purchased from Cell Signaling Technology (Denver, MA, USA). Mouse bax antibody was obtained from BD Transduction Laboratories (West Grove, PA, USA). Rabbit amyloid precursor protein (APP) antibody was purchased from MyBioSource (San Diego, CA, USA). Mouse monoclonal β-actin and rabbit polyclonal bcl-2 antibody were provided from Sigma-Aldrich Chemical. Horseradish peroxidase (HRP)-conjugated goat anti-rabbit IgG, goat anti-mouse and donkey anti-goat IgG were received from Jackson ImmunoResearch Laboratories (West Grove, PA, USA).

**Figure 1:**
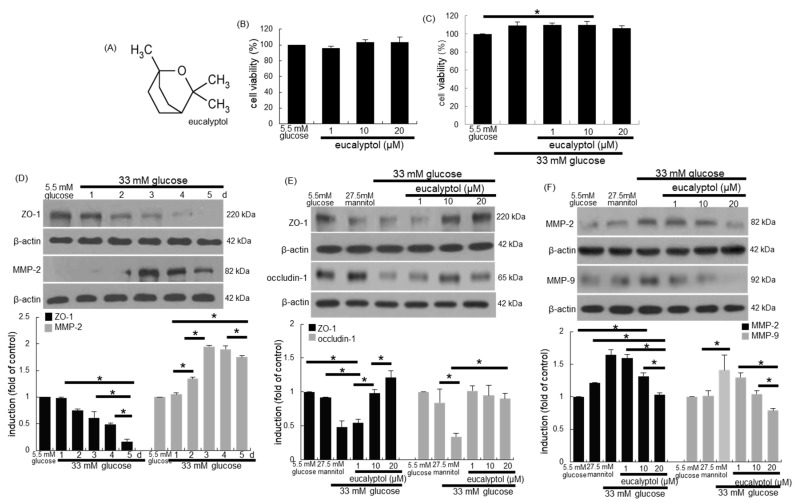
Chemical structure of eucalyptol (**A**), toxicity of eucalyptol-treated human retinal pigment epithelial (RPE) cells (**B**), inhibitory effects of eucalyptol on proliferation of glucose-exposed RPE cells (**C**), temporal responses of epithelial induction of ZO-1 and MMP-2 (**D**) and effects of eucalyptol on tight junction proteins of ZO-1, occludin-1, MMP-2 and MMP-9 injured by glucose (**E** and **F**). An MTT assay was conducted for measurement of cell viability (**B** and **C**, 100% viability with 5.5 mM glucose control). Bar graphs for viability (mean ± SEM, *n* = 5) was expressed as the percentage of cell survival, compared to the glucose control. Cell lysates were subject to western blot analysis with a primary antibody against ZO-1, occludin-1, MMP-2, MMP-9 and β-actin for a cellular internal control (**D**–**F**). Bar graphs (mean ± SEM, *n* = 3) in the panels represent densitometric results of blot bands. * Values in respective bar graphs indicate a significant difference at *p* < 0.05.

Eucalyptol (Figure 1A) was dissolved in dimethyl sulfoxide (DMSO, <0.5% concentration) for experiments.

### 2.2. RPE Cell Culture

Primary human RPE cells (Lonza) were cultured in DMEM containing 100 U/mL penicillin, and 100 µg/mL streptomycin at 37 °C humidified atmosphere of 5% CO_2_ in air. To mimic a hyperglycemic microenvironment, RPE cells were incubated in media containing 33 mM D-glucose in the absence and presence of 1–20 µM eucalyptol for up to 5 days. Additionally, RPE cells were cultured in media containing 5.5 mM D-glucose as a glucose control, or 27.5 mM mannitol (+5.5 mM D-glucose) as an osmotic control. In another set of experiments, RPE cells were incubated for 4 days in media containing 5 μM Aβ in the presence of 1–20 μM eucalyptol.

The RPE cell viability was determined with MTT (3-(4,5-dimethylthiazol-2-yl)-2,5- diphenyltertrazolium bromide) [30,31]. Eucalyptol at the doses of 1–20 µM did not show cytotoxicity (Figure 1B). The RPE cell growth was observed in 33 mM glucose-containing media for 4 days (Figure 1C). However, eucalyptol at 1–20 μM tended to lessen hyperglycemic proliferation.

### 2.3. In Vivo Animal Experiments

All the experiments were approved by the Committee on Animal Experimentation of Hallym University and conducted in compliance with the University’s Guidelines for the Care and Use of Laboratory Animals (hallymR1 2016-10).

Adult male db/db mice (C57BLKS/+Leprdb Iar; Jackson Laboratory, Sacramento, CA, USA) and their age-matched non-diabetic db/m littermates (C57BLKS/J; Jackson Laboratory, Sacramento, CA, USA) were introduced in the present study [30,31]. Mice were raised at 23 ± 1 °C with 50 ± 10% relative humidity (12 h light/12 h dark cycle) under specific pathogen-free conditions, and supplied with water ad libitum at the animal facility of Hallym University. Animals were divided into three subgroups (*n* = 9–10 for each subgroup), as described in previous studies [30,31]. The first group of mice was non-diabetic db/m control mice, and db/db mice were divided into two groups. One group of db/db mice was daily supplied 10 mg/kg BW eucalyptol via gavage for 8 weeks.

The fasting blood glucose levels and glycated hemoglobin were measured every other week from mouse tail veins during the 8 week-supplementation of eucalyptol [30]. The plasma insulin level was reduced in eucalyptol-challenged db/db mice [30].

### 2.4. Western Blot Analysis

Western blot analysis was carried out using whole cell lysates prepared in RPE cells (3.5 × 10^5^ cells/culture dish) and eye tissue extracts. Whole cell lysates and eye tissue extracts were prepared in a lysis buffer [30,31]. Cell lysates and tissue extracts containing equal amounts of proteins were electrophoresed on 8–15% SDS-PAGE and transferred onto a nitrocellulose membrane. Blocking nonspecific binding was achieved by using either 3% fatty acid-free bovine serum albumin (BSA) or 5% nonfat dry skim milk for 3 h. The membrane was incubated overnight at 4 °C with each primary antibody of the target proteins (dilution in 5% BSA, 1:1000) and washed in a Tris-buffered saline-Tween 20 for 10 min. The membrane was then incubated for 1 h with a secondary antibody conjugated to HRP (dilution in 3% BSA, 1:5000). Target protein levels were determined with immobilon Western chemiluminescent HRP substrate (Millipore, Merck KGaA, Darmstadt, Germany). Incubation with mouse monoclonal β-actin antibody was also conducted for comparison.

### 2.5. Hemotoxylin and Eosin (H&E) Staining

For the histological observation, eye tissue specimens were fixed in 4% buffered-formaldehyde. The paraffin-embedded eye tissue sections were deparaffinized and stained with H&E for 3 min and dehydrated in 95% alcohol. The H&E-stained tissue sections were observed using an optical microscope Axio Iimager system equipped with fluorescence illumination (Zeiss, Gottingen, Germany). Five images were captured for each tissue section.

### 2.6. RT-PCR Analysis

Total RNA was prepared from RPE cells using a Trizol reagent kit (Molecular Research Center, Cincinnati, OH, USA). The RNA (5 μg) was reversibly transcribed with 200 units of reverse transcriptase (Promega Co., Madison, WI, USA) and 0.5 mg/mL oligo-(dT)15 primer (Bioneer, Daejeon, Korea). The RT-PCR analysis was carried out for semi-quantifying the mRNA transcript levels of ZO-1 and MMP-2. The PCR condition for ZO-1 [5′-AGCCTGCAAAGCCAGCTCA-3′ (forward), 5′-AGTGGCCTGGATGGGTTCATAG-3′ (reverse, 104 bp)] and the condition for MMP-2 [5′-TGGCAAGTACGGCTTCTGTC-3′ (forward), 5′-TTCTTGTCGCGGTCGTAGTC-3′ (reverse, 180 bp)] were 94 °C (5 min), with 30 cycles at 94 °C (30 s), 55 °C (30 s) and 72 °C (30 s). The housekeeping gene of β-actin [5′-GACTACCTCATGAAGATC-3′ (forward), 5′-GATCCACATCTGCTGGAA-3′ (reverse, 500 bp)] was used for the co-amplification with respective gene.

### 2.7. ROS Generation

Dihydroethidium (DHE, Invitrogen, Carlsbad, CA, USA) staining was conducted for ROS production in RPE cells. RPE cells (0.5 × 10^4^ cells/slide) were fixed with 4% formaldehyde for 10 min and permeated by 0.1% Triton-X100 for 10 min on ice. RPE cells were stained by incubating with 20 μM DHE for 1 h. For the identification of nuclei, 4′,6-diamidino2-phenylindole (DAPI) was treated for 10 min. Stained cells on slides were mounted in a mounting solution. In addition, eye tissue specimens were fixed. The eye tissue sections (10 µm thickness) were stained with DHE. Slide images and stained tissue sections were photographed using an optical microscope system.

Oxidant generation in RPE cells was also assessed by staining of cell permeant reagent 2’,7’-dichlorofluorescin diacetate (DCF-DA). After challenge with 33 mM glucose, cells were loaded for 30 min with 10 μM DCF-DA in pre-warmed DMEM. After dye loading at 37 °C, the cells were washed thoroughly in phosphate-buffered saline, and the nuclear DAPI staining was performed. The culture images were photographed with an optical microscope system.

### 2.8. Immunocytochemical Staining

RPE cells (0.5 × 10^4^ cells/slide) were fixed with 4% formaldehyde for 10 min, permeated in 0.1% Triton-x 100 for 10 min on ice and blocked with a 4% FBS for 1 h. Immunofluorescent cytochemical staining of Aβ in RPE cells was performed using its primary antibody and FITC-conjugated IgG. DAPI was used for the nuclear staining. Images on slides were taken using an optical microscope system.

### 2.9. Hoechst 33258 Staining

After the fixation of RPE cells for 15 min on a glass-covered 24 well plates, 10 µg/mL Hoechst 33258 (Promega Co., Madison, WI, USA) was treated for the nuclear staining. Cells with fragmented or condensed nuclei were considered to be apoptotic. Each slide image was taken for detecting nuclear morphology with an optical microscope system.

### 2.10. Data Analysis

The data are presented as mean ± SEM for each treatment group in all the experiments. Statistical analyses were carried out using Statistical Analysis Systems (SAS Institute, Cary, NC, USA). Significance was evaluated by one-way analysis of variance and the following Duncan range test for multiple comparisons. Differences were regarded to be significant at *p* < 0.05.

## 3. Results

### 3.1. Inhibition of Glucose-Induced Loss of Epithelial Junction Proteins by Eucalyptol

Tight junctions enable the RPE monolayer to form the outer blood-retinal barrier that regulates solute transports between the fenestrated choroid capillaries and the photoreceptor layer of the retina [10,11]. The current study investigated that eucalyptol inhibited loss of RPE tight junction proteins under diabetic condition. The induction of the tight junction marker of ZO-1 was attenuated in RPE cells exposed to 33 mM glucose for 5 days in a temporal manner (Figure 1D). When 1–20 µM eucalyptol was added to glucose-loaded RPE cells for 4 days, the epithelial induction of ZO-1 and occludin-1 was highly reversed (Figure 1E).

The MMP proteins cause barrier disruption via aberrant proteolysis of epithelial tight junction proteins, leading to visual dysfunctions [13,32]. The induction of the MMP-2 was temporally enhanced in glucose-loaded RPE cells (Figure 1D). In contrast, the glucose-induced expression of epithelial MMP-2 and MMP-9 was dose-dependently attenuated by treating eucalyptol to RPE cells (Figure 1F). Accordingly, eucalyptol may block barrier disruption in diabetic retina through dampening the MMP induction.

### 3.2. Blockade of Diabetic Disruption of Blood-Retinal Barrier by Eucalyptol

**Figure 2:**
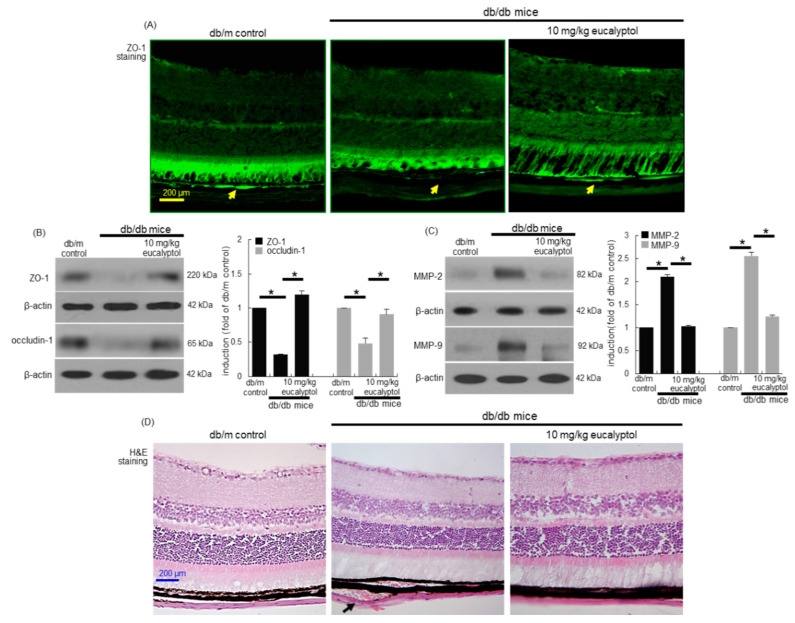
Immunohistochemical data (**A**) and western blot data (**B** and **C**) showing eye tissue levels of ZO-1, occludin-1, MMP-2 and MMP-9 in eucalyptol-supplemented db/db mice. For the immunohistochemical analysis of ZO-1, green FITC-conjugated secondary antibody was used for visualizing ZO-1 induction (**A**). Yellow arrows indicate retinal pigment epithelial ZO-1. Tissue extracts were subject to western blot analysis with a primary antibody against ZO-1, occludin-1, MMP-2, MMP-9 and β-actin for an internal control (**B** and **C**). The bar graphs (mean ± SEM, *n* = 3) represent quantitative results of blots in the panels. * Values in respective bar graphs indicate a significant difference at *p* < 0.05. Inhibition of retinal pigment epithelial detachment from Bruch’s membrane by eucalyptol in db/db mice (**D**, black arrow). Histological sections of mouse retina were H&E-stained. Each microphotograph is representative of four mice (**A** and **D**). Scale bar: 200 μm.

Aberrant alterations of the blood-retinal barrier are involved in the development of macular edema [4,6]. Consistent with the RPE cell culture results (Figure 1E,F), the in vivo animal data supported that eucalyptol inhibited diabetic disruption of cell-to-cell tight junction. The FITC-immunohistochemical data revealed that the ZO-1 induction (yellow arrows) declined in diabetic retinal tissues of mice (Figure 2A). Oral administration of 10 mg/kg eucalyptol restored the ZO-1 induction reduced in diabetic retinal tissues (Figure 2A). Consistently, the western blot data showed that eucalyptol induced the retinal expression of ZO-1 and occludin-1 in diabetic animals (Figure 2B). When 10 mg/kg eucalyptol was orally supplemented to db/db mice, the induction of MMP-2 and MMP-9 was diminished (Figure 2C). Thus, eucalyptol may block the breakdown of blood-retinal barrier of RPE.

Histological alterations of RPE are major factors causing disruption of ocular malfunction of visual cycle in DR [33]. The detachment of Bruch’s membrane from RPE cells was observed in eye tissues of db/db mice, evidenced by the histological H&E staining (Figure 2D). However, oral administration of 10 mg/kg eucalyptol apparently inhibited such pathological detachment. Accordingly, eucalyptol may ameliorate diabetic degeneration of RPE and Bruch’s membrane for the robust blood-retinal barrier.

### 3.3. Anti-Apoptotic Role of Eucalyptol in Diabetic Rpe Cells and Retina

**Figure 3:**
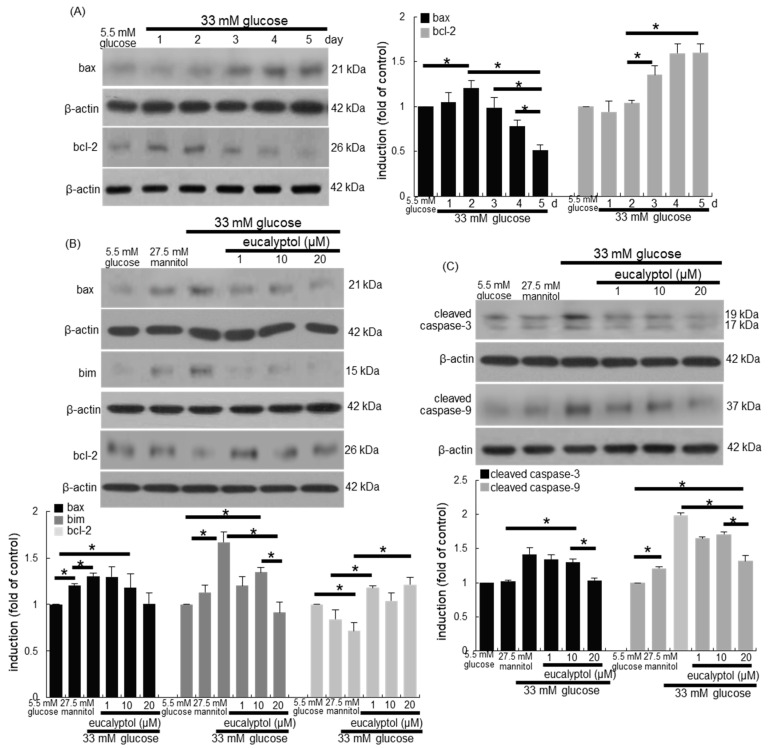
Time course responses of induction of bax and bcl-2 (**A**), and reciprocal effects of eucalyptol on induction of bax, bim, bcl-2, cleaved caspase-3 and cleaved caspase-9 (**B** and **C**). RPE cell lysates were subject to western blot analysis with a primary antibody against bax, bcl-2, bim, cleaved caspase-3, cleaved caspase-9 and β-actin as an internal control. The bar graphs (mean ± SEM, *n* = 3) represent quantitative results of blots in the panels. * Values in respective bar graphs indicate a significant difference at *p* < 0.05.

This study examined whether apoptosis of glucose-loaded RPE cells was responsible for loss of epithelial tight junction proteins. In RPE cells exposed to 33 mM glucose, the induction of pro-apoptotic bax and anti-apoptotic bcl-2 was temporally reciprocal (Figure 3A). The induction of bax and bim by glucose was reduced by treating 1–20 μM eucalyptol to RPE cells for 4 days (Figure 3B). On the contrary, the reduced bcl-2 induction was restored in eucalyptol-added RPE cells exposed to glucose (Figure 3B). In addition, the activation of caspase-3 and caspase-9 was enhanced in diabetic RPE cells, which was highly attenuated in 20 μM eucalyptol-treated RPE cells (Figure 3C).

**Figure 4:**
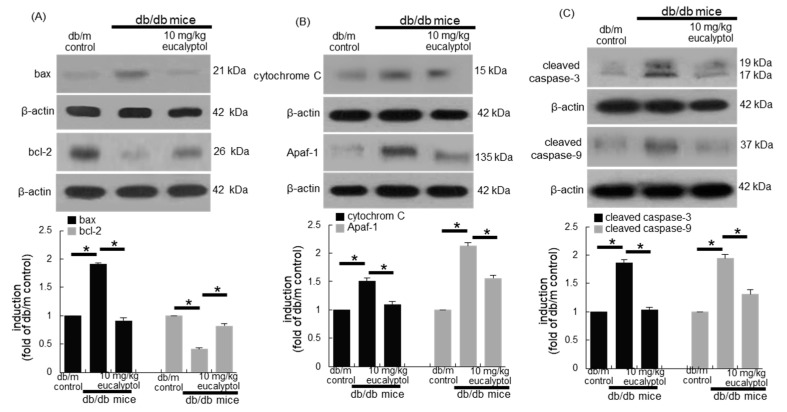
Inhibitory effects of eucalyptol on retinal apoptosis in diabetic eyes. Retinal cortical tissue extracts were subject to western blot analysis with a primary antibody against bax, bcl-2, cytochrome C, Apaf-1, cleaved caspase-3, cleaved caspase-9 and β-actin for an internal control (**A–C**). The bar graphs (mean ± SEM, *n* = 3) represent the quantitative results of blots in the panels. * Values in respective bar graphs indicate a significant difference at *p* < 0.05.

This study further confirmed that retinal epithelial apoptosis led to diabetes-associated injury of epithelial tight junction and blood-retinal barrier. As expected, the tissue bax level was elevated in diabetic eyes, while the bcl-2 level was abolished (Figure 4A). However, the induction of these apoptosis-related proteins was reciprocally restored by supplementing 10 mg/kg eucalyptol to diabetic mice. Consistently, eucalyptol reduced the expression of mitochondrial apoptotic proteins of cytochrome C and Apaf-1 elevated in diabetic eyes (Figure 4B). Furthermore, the activation of both caspase-3 and caspase-9 in eye tissues of db/db mice were diminished by providing 10 mg/kg eucalyptol to these animals (Figure 4C).

### 3.4. Suppression of Glucose-Induced Oxidative Stress by Eucalyptol

**Figure 5:**
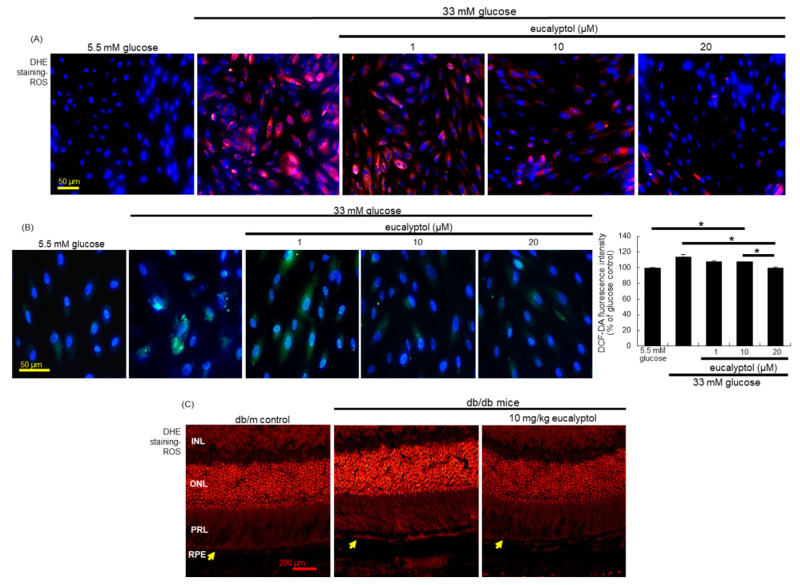
Inhibition of ROS production by eucalyptol in RPE cells (**A** and **B**) and in retina (**C**). For the measurement of ROS production, the DHE staining was conducted in RPE cells (**A**) and in retina (**C**). INL, inner nuclear layer; ONL, outer nuclear layer; PRL, photo receptor layer; RPE, retinal pigment epithelium monolayer. The yellow arrows indicate RPE layer. Furthermore, the DCF-DA staining for ROS production (**B**) were carried out in eucalyptol-treated RPE cells, and the DCF staining intensity (mean ± SEM, *n* = 3) was measured. Nuclear staining was done with 4′,6-diamidino-2-phenylindole. DHE- or DCF-stained RPE cells or retina were visualized under fluorescent microscopy. Scale bars: 50–200 μm. * Bar graph values indicate a significant difference at *p* < 0.05.

This study examined whether eucalyptol inhibited ROS generation in glucose-exposed RPE cells. Strong red-fluorescent DHE-responsive superoxides and H_2_O_2_ was highly produced in glucose-exposed RPE cells (Figure 5A). However, 1–20 μM eucalyptol diminished the production of these oxidants in RPE cells by glucose. In RPE cells treated with 1–20 μM eucalyptol, there was a reduction in green-fluorescent staining of DCF-DA observed (Figure 5B). Additionally, the DCF-dependent fluorescence intensity was enhanced in glucose-loaded RPE cells, showing that glucose induced intracellular H_2_O_2_-derived oxidative stress (Figure 5B). Accordingly, retinal epithelial apoptosis may be attributed to glucose-induced intracellular ROS and oxidative stress. Furthermore, the DHE fluorescence in RPE/choroid was markedly attenuated in diabetic mice that received 1–20 μM eucalyptol (yellow arrows, Figure 5C), suggesting that eucalyptol improved barrier integrity of RPE through inhibiting superoxide generation in RPE/choroid.

### 3.5. Inhibitory Effects of Eucalyptol on Glucose-Induced aβ Formation

**Figure 6:**
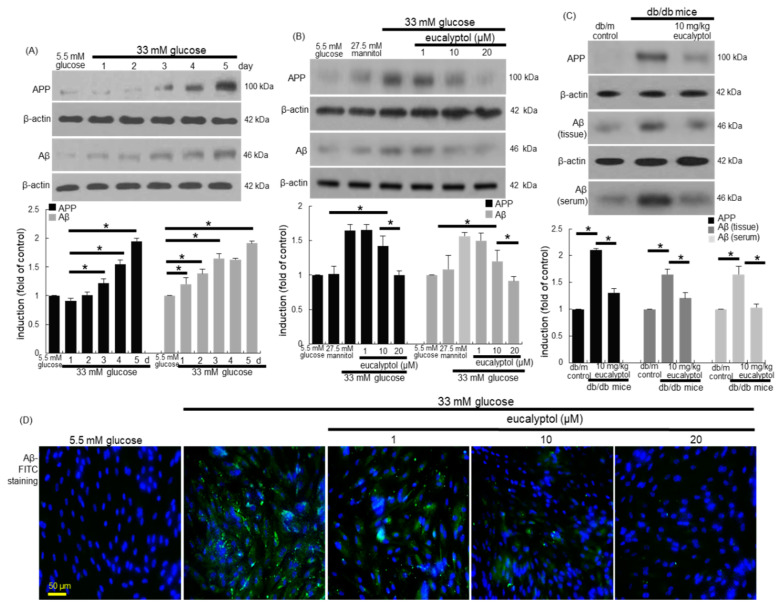
Temporal induction of APP and Aβ (**A**) and the inhibitory effects of eucalyptol on their induction (**B**–**D**). RPE cell lysates, eye tissue extracts and sera were subject to western blot analysis with a primary antibody against APP, Aβ, and β-actin for an internal control. The bar graphs (mean ± SEM, *n* = 3) represent quantitative results of blot bands (**A–C**). * Values in respective bar graphs indicate a significant difference at *p* < 0.05. For the immunocytochemical analysis of Aβ, green FITC-conjugated secondary antibody was used for visualizing Aβ induction, being counterstained with 4′,6-diamidino-2-phenylindole for the nuclear staining (**D**). Each microphotograph (mean ± SEM, *n* = 3) was obtained by using a microscope system. Scale bar: 50 μm.

Based on direct evidence that diabetes mellitus causes an increase in the Aβ peptide levels [20,21], this study examined whether glucose per se induced Aβ formation in RPE cells, which was deterred by treating eucalyptol. When RPE cells were exposed to 33 mM glucose for 5 days, the cellular levels of APP and Aβ were temporally prompted, with 50–60% increase of these proteins by glucose for 4 days (Figure 6A). However, such an increase was reversed by treating 1–20 μM eucalyptol, in which the cellular levels of APP and Aβ were significantly reduced in a dose-dependent manner (Figure 6B). In addition, western blot data revealed that eye tissue levels of APP and Aβ were elevated in diabetic mice (Figure 6C). When 10 mg/kg eucalyptol was orally given to these animals for 8 weeks, the induction of these proteins was diminished in db/db mouse eyes. Furthermore, the serum Aβ level enhanced in diabetic mice was reduced by supplying 10 mg/kg eucalyptol to the animals (Figure 6C).

The FITC-green staining data of Aβ confirmed that eucalyptol reduced the epithelial induction of Aβ enhanced by glucose (Figure 6B). There was weak cytoplasmic green-staining in RPE cells under glucose control condition, while the 4 day-glucose stimulation caused a strong FITC staining of Aβ (Figure 6D). Thus, eucalyptol may inhibit diabetes-associated RPE amyloidosis, ultimately leading to disruption of the blood-retinal barrier.

### 3.6. Suppressive Effects of Eucalyptol on aβ-Induced Disruption of Tight Junctions

**Figure 7:**
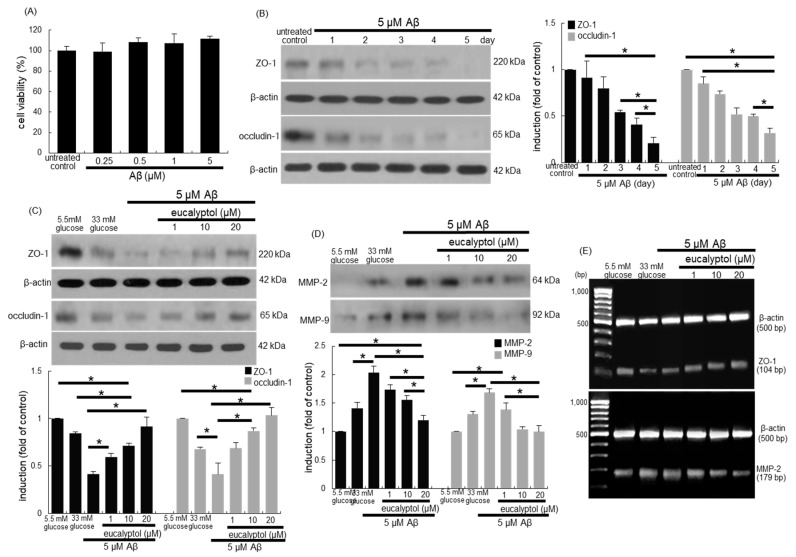
Dose-responses of Aβ to human retinal pigment epithelial (RPE) cell toxicity (**A**), temporal induction of tight junction markers of ZO-1 and occludin-1 by Aβ (**B**) and effects of eucalyptol on cellular induction of ZO-1 and occludin-1 and secretion of MMP-2 and MMP-9 (**C** and **D**). Cell viability was measured by using an MTT assay (A, 100% viability with untreated control). Bar graphs for viability (mean ± SEM, *n* = 5) was expressed as percent cell survival. RPE cell lysates were subject to western blot analysis using a primary antibody of ZO-1, occludin-1, MMP-2, MMP-9 and β-actin for an internal control (**B**–**D**). The bar graphs (mean ± SEM, *n* = 3) represent quantitative results of blot bands. * Values in respective bar graphs indicate a significant difference at *p* < 0.05. The mRNA transcriptional levels of ZO-1 and MMP-2 were measured using RT-PCR analysis. β-Actin was used as a housekeeping gene for the co-amplification with ZO-1 and MMP-2 (**E**).

When 0.25–5 μM Aβ was loaded to RPE cells, no noticeable RPE cytotoxicity was observed (Figure 7A). The induction of ZO-1 and occludin-1 was gradually diminished in 5 µM Aβ-loaded RPE cells in a temporal manner (Figure 7B). In contrast, 1–20 µM eucalyptol dose-dependently prompted the expression of these proteins dampened by 5 μM Aβ (Figure 7C). On the other hand, this study found that the secretion of MMP-2 and MMP-9 was upregulated in Aβ-exposed RPE cells, which was reduced by adding 1–20 µM eucalyptol to these cells (Figure 7D). Furthermore, nontoxic eucalyptol enhanced the ZO-1 transcription reduced by Aβ, indicating that the ZO-1 induction by eucalyptol was regulated at its transcriptional levels (Figure 7E). In contrast, the transcriptional level of MMP-2 elevated by Aβ was downregulated by the presence of eucalyptol in a dose-dependent manner.

### 3.7. Blockade of aβ-Induced Apoptosis by Eucalyptol

**Figure 8:**
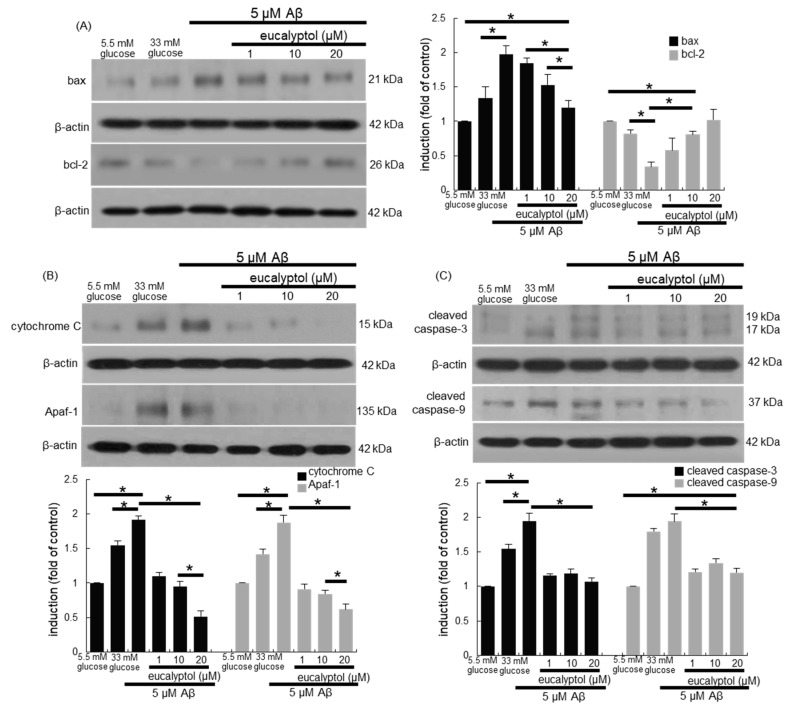
Blockade of Aβ-induced apoptosis by eucalyptol in human retinal pigment epithelial (RPE) cells. RPE cell lysates were subject to western blot analysis with a primary antibody against bax, bcl-2, cytochrome C, Apaf-1, cleaved caspase-3, cleaved caspase-9 and β-actin for an internal control (**A**–**C**). Bar graphs (mean ± SEM, *n* = 3) in the panels represent densitometric results of blot bands. * Values in respective bar graphs indicate a significant difference at *p* < 0.05.

This study examined whether Aβ stimulated apoptotic death of RPE cells, which was inhibited by supplementing eucalyptol. Consistent with 33 mM glucose-loading data (Figure 3A), Aβ influenced the expression of bax and bcl-2 proteins in RPE cells (Figure 8A). However, 1–20 µM eucalyptol dose-dependently restored the induction of anti-apoptotic bcl-2 dampened by amyloid-β, while the cellular level of pro-apoptotic bax was reduced in eucalyptol-supplied RPE cells. In addition, treating RPE cells with eucalyptol reduced the induction of cytochrome C and Apaf-1 by Aβ (Figure 8B). Furthermore, Aβ highly activated caspase-3 and caspase-9 in RPE cells, which was significantly diminished in ≥1 μM eucalyptol-treated RPE cells (Figure 8C).

**Figure 9:**
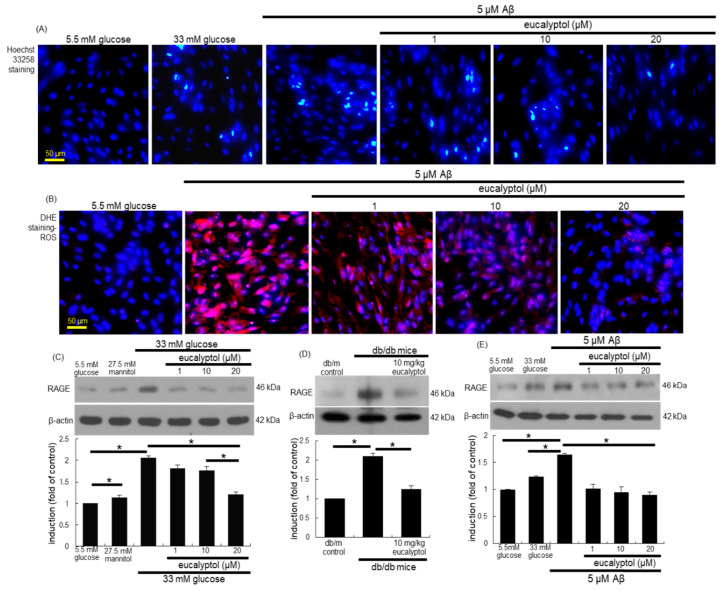
Blockade of Aβ-induced nuclear condensation (**A**), cellular ROS production (**B**) and cellular RAGE induction (**C**–**E**) by eucalyptol in human retinal pigment epithelial (RPE) cells. Hoechst 33258 staining was done for the measurement of nuclear condensation of RPE cells exposed to 5 µM Aβ (**A**). For the measurement of ROS production (**B**), DHE staining was conducted in eucalyptol-treated RPE cells. Nuclear staining was done with 4′,6-diamidino-2-phenylindole. Hoechst 33258- and DHE-stained RPE cells were visualized under fluorescent microscopy. Each microphotograph (mean ± SEM, *n* = 3) was obtained by using a microscope system. Scale bars: 50 μm. RPE cell lysates were subject to western blot analysis with a primary antibody against RAGE, and with β-actin as an internal control (**C**–**E**). Bar graphs (mean ± SEM, *n* = 3) in the panel represent densitometric results of blot bands. * Values in respective bar graphs indicate a significant difference at *p* < 0.05.

Hoechst 33258 staining revealed that 5 µM Aβ highly elicited nuclear condensation and fragmentation of RPE cells, proving that Aβ promoted RPE cell apoptosis (Figure 9A). In contrast, 1–20 µM eucalyptol blocked Aβ-induced nuclear condensation of RPE cells. To determine whether Aβ-induced retinal epithelial apoptosis entailed ROS generation, superoxide production was detected as DHE fluorescence in RPE cells exposed to Aβ for 4 days. There was strong DHE fluorescence in 5 μM Aβ-loaded RPE cells (Figure 9B). However, the supply of ≥1 µM eucalyptol to RPE cells attenuated cellular ROS generation.

### 3.8. Blockade of Aβ-RAGE Interaction by Eucalyptol

There is much evidence that the AGE-RAGE-oxidative stress axis place a role in diabetic vascular complications [34]. We found that glucose strongly enhanced AGE formation in RPE cells (unpublished data). RAGE is a multi-ligand receptor capable of binding diverse molecules including AGE and Aβ [35,36]. High glucose elevated the RAGE induction in RPE cells (Figure 9C). In contrast, 1–20 μM eucalyptol attenuated the retinal epithelial induction of RAGE enhanced by glucose. In addition, the eye tissue level of RAGE was elevated in db/db mice (Figure 9D). When diabetic animals were administered with 10 mg/kg eucalyptol, the tissue level of RAGE was reduced. As expected, the RAGE induction was promoted in Aβ-loaded RPE cells. When RPE cells were treated with ≥1 µM eucalyptol, the epithelial induction of RAGE by Aβ was highly abrogated (Figure 9E).

## 4. Discussion

**Figure 10:**
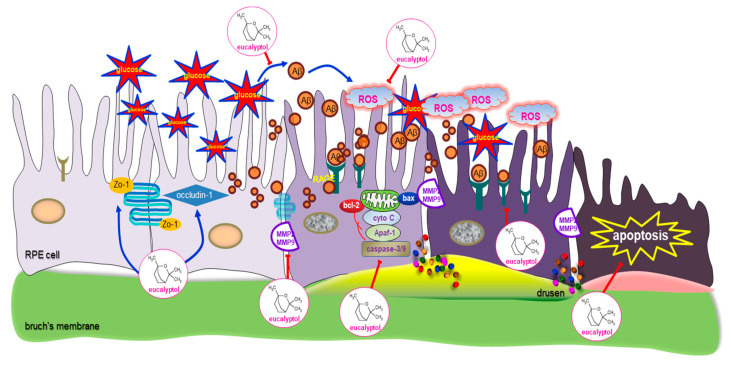
Schematic figure outlining the inhibition of epithelial tight junction disruption by eucalyptol following exposure of glucose and Aβ to human retinal pigment epithelial (RPE) cells.

Nine major findings were obtained from this study. (1) Nontoxic eucalyptol lessened the induction of APP and Aβ elevated in 33 mM glucose-loaded RPE cells and in db/db mouse eyes. (2) Eucalyptol restored the induction of the tight junction proteins of ZO-1 and occludin-1 in RPE cells diminished by glucose and 5 µM Aβ. (3) Eucalyptol attenuated the induction of MMP-2 and MMP-9 in RPE cells by glucose and 5 µM Aβ. (4) Oral administration of 10 mg/kg eucalyptol restored retinal tissue levels of ZO-1, occludin-1, MMP-2 and MMP-9 in diabetic mice, accompanying the inhibition of detachment of RPE from Bruch’s membrane. (5) While treating 1–20 μM eucalyptol to RPE cells reduced glucose- and Aβ-induced pro-apoptotic proteins of bax, bim and cleaved caspase-3 and cleaved caspase-9, the anti-apoptotic bcl-2 was upregulated in eucalyptol-treated RPE cells stimulated by glucose or Aβ. (6) Supplementing eucalyptol to diabetic mice reciprocally reversed retinal tissue levels of apoptosis-related proteins of bcl-2, bax, cytochrome C, Apaf-1 caspase-3 and caspase-9. (7) Eucalyptol attenuated the ROS generation in glucose- or Aβ-exposed RPE cells and in diabetic RPE. (8) Submicromolar eucalyptol inhibited Aβ-elicited nuclear condensation and fragmentation of RPE cells. (9) Eucalyptol attenuated the RPE induction of RAGE enhanced by glucose and Aβ and also in db/db mouse eyes. All together, these results indicated that eucalyptol may ameliorate RPE injury and barrier integrity in diabetic retina through combating Aβ-triggered ROS generation and RAGE induction (Figure 10).

The RPE is situated between neuro-retina and choroid capillaries, where it forms the outer blood-retinal barrier. Dynamic barrier functions of RPE are crucial for maintaining optimal retinal health [1,3,4]. The tight junctions involving ZO-1 and occludin-1 proteins between neighboring RPE cells are an integral structural component of the blood-retinal barrier [4,11]. Loss of RPE cell to cell junction and impairment of blood-retinal barrier are typical events in retinal degenerative diseases, resulting in vascular leakage and retinal edema in DR and RP [2,7,8]. Furthermore, the RPE glucose transport to photoreceptors is diminished in RP, and metabolic deregulation of the blood-outer retinal barrier occurs [5]. Accordingly, the maintenance of the RPE integrity is vital in the visual functions. This study found that the levels of ZO-1 and occludin-1 were greatly reduced in diabetic RPE cells and in db/db mouse eyes. In addition, the MMP proteins of RPE were upregulated in glucose-loaded RPE cells and in diabetic mouse eyes. Elevated expression of MMP proteins in the retina increases vascular permeability by a mechanism related to proteolytic decomposition of the tight junction proteins [13]. A study reported that MMP-2-mediated degradation of occludin contributes to blood-brain barrier damage in early ischemic stroke [37]. In this study, the H&E staining showed that hyperglycemia resulted in an increase in separation between the RPE and the Bruch’s membrane in retina. On the other hand, several retinal cells experienced cell death in a diabetic environment [38]. This study showed that glucose instigated oxidative stress-mediated apoptotic signaling in RPE cells. In addition, hyperglycemia stimulated apoptotic bax signaling, mitochondrial pathways of cytochrome C/Apaf-1 apoptosomes and proteolytic caspase activation in mouse eyes. Similarly, diabetic insult of high glucose promotes mitochondrial pathways of cell apoptosis in RPE by regulating ROS-mediated inhibition of mitophagy [39]. A recent report highlights the consequence of autophagy and oxidative stress as two related mechanisms involved in RP [40]. Identifying mechanisms of cell death will lead to a more targeted approach in the development of new therapies to treat DR and RP. Several retina pathologies, such as DR and secondary cone photoreceptor death in RP, may entail mitochondrial dysfunction [40].

Several studies have shown that naturally-occurring compounds inhibit oxidative stress-induced RPE cell damage through enhancing antioxidant activity and anti-apoptotic function [41,42]. For both RP and DR, botanical compounds and plant extracts may have significant effects on treatment and prevention [43]. Efficient treatment of RP can be antioxidant agents and autophagy modulators, which curtails the excessive ROS levels and regulates autophagy [40]. Diphlorethohydroxycarmalol and kaemperol protect RPE cells from oxidative stress-induced DNA damage and apoptosis [41,42]. Curcumin inhibits N-methyl-N-nitrosourea-induced photoreceptor cell apoptosis by attenuating DNA oxidative stress, indicating that curcumin may inhibit the onset and progression of human RP [44]. This study revealed that eucalyptol suppressed ROS generation and modulated apoptosis-related protein induction in RPE cells and eye tissues in parallel with cytochrome C/Apaf-1 apoptosome formation and proteolytic caspase activation under the glucotoxic condition. Accordingly, eucalyptol blocked hyperglycemia-triggered oxidative stress-mediated RPE apoptosis through disturbing the bax-caspase signaling pathway. Furthermore, eucalyptol ameliorated the glucose-damaged barrier function of RPE by restoration of loss of the tight junction-associated proteins of ZO-1 and occludin-1 and by inhibition of RPE detachment from Bruch’s membrane. Thus, it can be assumed that oxidative stress may be one of the pathways implicated in RPE barrier dysfunction and retinal degeneration promoted by glucose. However, whether the primary target of oxidative stress by hyperglycemia in DR is RPE cells and/or photoreceptors remains unclear. Retinal Müller cells are shown to undergo inflammation-driven cell death in the diabetic retina [38]. Considering that DR and RP are chronic inflammatory diseases [43], retinal inflammation-induced cell death might play an important role in the RPE barrier dysfunction. The RPE dysfunction in age-related macular degeneration entails the NLRP3 inflammasome activation induced by Aβ-responsive oxidative stress [26]. Unfortunately, this study did not examine whether glucose-induced retinal inflammation was responsible for RPE barrier dysfunction. It is shown that eucalyptol exerts anti-inflammatory activity [27].

A rich literature describes how hyperglycemia affects RPE-specific barrier functions [6,8]. Recent work identifies possible mechanisms for new targets and therapeutic strategies to reverse retinal dysfunction and blood-derived content leakage in DR and RP [5,45]. However, the underlying mechanisms still remain to be elucidated. Accumulating epidemiologic evidence suggests that people with diabetes mellitus are at increased risk of Alzheimer’s disease [46,47]. Neurodegenerative diseases such as Alzheimer’s disease and RP induce spontaneous cell death via same basic cell death mechanisms [17]. Several studies have reported a potential mechanistic linking between diabetes mellitus and increase in Aβ peptide levels [20,21]. A proteomic study reveals that amyloid processing pathways are enhanced in diabetic retinas without glial activation [48]. Thus, it was necessary to elucidate the relationship between hyperglycemia and Aβ accumulation in diabetic retina. This study found that APP and Aβ were induced in diabetic RPE cells and diabetic eyes, possibly contributing to retinal cell apoptosis and loss of tight junction proteins. Eucalyptol is reported to reduce Aβ-induced inflammation in differentiated PC12 cells and to act as an anti-inflammatory agent in neurodegenerative diseases including Alzheimer’s disease [49]. The treatment of Aβ-induced impairment of RPE barrier with eucalyptol led to a new target and therapeutic strategy during glucotoxic conditions. One investigation shows that hyperglycemia inhibits APP degradation and enhances Aβ production via a reduction of APP turnover rate [50]. In fact, eucalyptol suppressed the generation of APP and Aβ and the RAGE induction in glucose-loaded RPE cells, and reduced the Aβ level locally in eyes and systemically in blood. Accordingly, it can be speculated that eucalyptol may increase Aβ degradation and elimination.

In summary, this study attempted to determine the capability of eucalyptol in counteracting Aβ-mediated malfunction of RPE tight junction and barrier in glucose-loaded RPE and diabetic mouse eyes. Oral supplementation of eucalyptol inhibited the proteolytic MMP induction in diabetic eyes, thus maintaining a strong RPE adhesion to Bruch’s membrane. Nontoxic eucalyptol deterred glucose-mediated Aβ-induced loss of cell to cell junction proteins of RPE in parallel with inhibition of RAGE induction. Furthermore, eucalyptol inhibited the generation of APP and Aβ in diabetic retina, consequently leading to oxidative stress and caspase-dependent RPE apoptosis. Therefore, eucalyptol may be a potent retinoprotective agent ameliorating diabetes-associated blood-retinal barrier disruption via inhibition of Aβ-mediated oxidative stress-dependent apoptotic signaling pathway. The amyloid plaques in brains of patients with Alzheimer’s disease resemble the amyloid deposits in pancreatic islet of diabetic patients with decreased β-cell mass [51]. Elevated insulin level in diabetes promotes Aβ accumulation by competing with Aβ for insulin-decomposing enzyme [51,52]. Although further studies are needed, compromised glucose metabolism and insulin signaling in diabetic eyes may be attributed to Aβ accumulation and RPE barrier dysfunction.
